# Inhibition of CDK9 alleviates osteoarthritis by suppressing inflammation and reducing chondrocyte apoptosis

**DOI:** 10.3389/fphar.2026.1784174

**Published:** 2026-03-11

**Authors:** Bo Zhang, Zebin Wu, Wentao Wang, Hao Xu, Yuhu Zhao, Po Zhang, Yaozeng Xu

**Affiliations:** 1 Department of Orthopedics, Orthopedic Institute, Medical College, The First Affiliated Hospital of Soochow University, Soochow University, Suzhou, Jiangsu, China; 2 Department of Orthopaedics, Affiliated Hospital of Nantong University, Medical School of Nantong University, Nantong, Jiangsu, China; 3 Department of Orthopedics, The Affiliated Sihong First People’s Hospital of Xuzhou Medical University, Xuzhou Medical University, Suqian, Jiangsu, China

**Keywords:** CDK9, flavopiridol, inflammation, NF-κB, posttraumatic osteoarthritis

## Abstract

**Background:**

Posttraumatic osteoarthritis (PTOA) develops following joint trauma and leads to pain and functional impairment. This study aimed to investigate the role and mechanism of cyclin-dependent kinase 9 (CDK9) in PTOA progression.

**Methods:**

We established an *in vitro* PTOA model by treating primary rat chondrocytes with lipopolysaccharide (LPS). CDK9 expression was modulated using siRNA, overexpression plasmids, and the inhibitor flavopiridol (FLA). Inflammatory cytokines were quantified by ELISA. RT-qPCR and Western blot were used to assess CDK9 and NF-κB pathway components. Cell viability and apoptosis were measured by CCK-8 assay and flow cytometry, respectively. An *in vivo* PTOA rat model was generated by medial meniscectomy (MMx). Rats received intra-articular injections of FLA (1, 3.5, 7.5 mg/kg) or IL-1β. Histopathological changes were evaluated by hematoxylin-eosin (HE) and Safranin O-Fast Green staining.

**Results:**

*In vitro*, FLA and si-CDK9 attenuated LPS-induced inflammation and apoptosis, enhanced cell viability, and suppressed CDK9/NF-κB activation. Conversely, CDK9 overexpression exacerbated these detrimental effects. *In vivo*, FLA treatment (particularly at 7.5 mg/kg) significantly inhibited CDK9 and NF-κB activation in articular cartilage, improved mechanical withdrawal threshold and locomotor activity, reduced cartilage degradation, and lowered levels of TGF-β, IL-6, TNF-α, and IL-1β. However, co-administration of IL-1β, an NF-κB activator, reversed the therapeutic effects of FLA in both models.

**Conclusion:**

Our findings demonstrate that CDK9 plays a critical role in PTOA pathogenesis. Inhibition of CDK9 alleviates disease progression by suppressing the NF-κB signaling pathway, highlighting its potential as a therapeutic target for PTOA.

## 1 Introduction

With the global aging population, the prevalence of osteoarthritis (OA) continues to rise. Posttraumatic osteoarthritis (PTOA), which accounts for approximately 12% of all OA cases, develops in 20%–50% of patients following joint trauma (Wang et al., 2020; Maia et al., 2023; Cinque et al., 2018). As a common and prototypical form of OA, PTOA involves the degeneration of articular cartilage and pathological alterations in associated structures such as the synovium, joint capsule, ligaments, and subchondral bone, ultimately leading to joint pain and functional impairment (Safiri et al., 2020; Huang et al., 2022b). PTOA predominantly affects weight-bearing joints, including the ankle, knee, shoulder, and hip (Herrera-Perez et al., 2022; Hunter and Bierma-Zeinstra, 2019).

The primary risk factors for PTOA include disruption of articular surface integrity due to trauma (e.g., intra-articular fractures) (Driban et al., 2014; Sward et al., 2014), as well as congenital deformities, malunion, or angular deformities following fractures (Wellsandt et al., 2016). Despite these known associations, the precise pathogenesis of PTOA remains incompletely understood. Multiple secondary mechanisms contribute to disease progression after joint injury, including direct cartilage damage and degeneration (Desai et al., 2022; Sebastian et al., 2021; Duan et al., 2021), a heightened inflammatory milieu driven by local cytokines (Song et al., 2021; Bedingfield et al., 2021; Mao et al., 2024), oxidative stress from excessive reactive oxygen species (ROS) production (Kang et al., 2022; Yagi et al., 2023), and abnormal mechanical loading (Zhang et al., 2022; Logerstedt et al., 2022).

Cyclin-dependent kinases (CDKs), a family of serine/threonine protein kinases, play crucial roles in regulating cell cycle progression and transcriptional processes through substrate phosphorylation (Malumbres, 2014). Among the twenty-one identified CDKs, dysregulated expression of several is frequently associated with cancer, leading to the development of CDK inhibitors primarily for oncological therapy. However, only a few have gained clinical approval (Fassl et al., 2022; Shi et al., 2022). Current selective inhibitors mainly target CDK1, CDK2, CDK4/6, CDK7, and CDK9 (Tadesse et al., 2020), functioning by disrupting cyclin-CDK interactions to impede cell cycle progression (Li et al., 2022; Ning et al., 2022).

CDK9, distinct from other CDKs, does not regulate the cell cycle. It serves as the kinase component of positive transcription elongation factor b (P-TEFb), promoting transcriptional elongation (Alsfouk, 2021; Fujinaga et al., 2023). CDK9 also interacts with various transcription factors to regulate anti-apoptotic protein expression, thereby influencing cell survival (Egloff, 2021). Notably, CDK9 is frequently dysregulated in malignancies and represents a promising target for cancer therapy (Huang Z. et al., 2022; Mandal et al., 2021). Despite its established role in oncology, research on CDK9 in PTOA is limited. A few studies suggest that CDK9 inhibition may ameliorate arthritis (Fukui et al., 2021; Xue et al., 2019), yet the underlying mechanisms in PTOA are unclear.

Given the emerging significance of CDK9 in various pathologies and its underexplored role in PTOA, this study aims to investigate the effects of CDK9 modulation on PTOA progression. Using both *in vitro* and *in vivo* models, we evaluated the therapeutic potential of the CDK9 inhibitor flavopiridol (FLA) and explored the involvement of the NF-κB signaling pathway (Joshi et al., 2023; Yasuda, 2011). Our findings elucidate a novel molecular mechanism by which CDK9 influences PTOA and highlight CDK9 inhibition as a potential therapeutic strategy.

## 2 Methods

### 2.1 Network pharmacology analysis

The 2D chemical structure and 3D conformation of FLA were obtained from the PubChem database (https://pubchem.ncbi.nlm.nih.gov/) with the PubChem Compound Identifier (CID) 44297210. Microarray dataset GSE51588 was downloaded from the GEO database (https://www.ncbi.nlm.nih.gov/geo/), containing genome-wide expression profiles of human osteoarthritis (OA) subchondral bone. Differential expression analysis between OA and normal samples was performed using the R package limma with default parameters. Genes with |log_2_ (fold change)| > 1 and adjusted P-value <0.05 were considered significantly differentially expressed. Genes with log_2_FC > 1 and adjusted P < 0.05 were defined as upregulated, while those with log_2_FC < −1 and adjusted P < 0.05 were defined as downregulated. The results were visualized as a heatmap and a volcano plot.

The STITCH database (http://stitch.embl.de/) was used to predict potential target proteins of flavopiridol (FLA) with a confidence score threshold of 0.7 (a commonly used high-confidence threshold) to ensure the reliability of predicted targets. Protein-protein interaction (PPI) networks between FLA targets and components of the NF-κB signaling pathway were analyzed using the STRING database (https://string-db.org/) with the following critical parameters: minimum interaction score of 0.7 (high confidence), hiding disconnected nodes in the network, and setting the maximum number of interactors to 500. Additionally, topological parameters including degree centrality and betweenness centrality were calculated to identify hub proteins in the PPI network, which are critical for maintaining network stability and function. Molecular docking between FLA and CDK9 (PDB ID: 3BLQ) was performed using AutoDock Vina 1.2.2, with binding affinity expressed as kcal/mol.

### 2.2 Isolation, culture, and identification of chondrocytes

Articular cartilage was aseptically harvested from the knee joints of five 4-week-old Sprague-Dawley (SD) rats. Tissues were minced and digested sequentially with 0.25% trypsin-EDTA for 30 min and 0.2% type II collagenase in DMEM for 2 h at 37 °C. Isolated chondrocytes were cultured in RPMI-1640 medium supplemented with 10% fetal bovine serum (FBS, Gibco, United States), 100 U/mL penicillin, and 100 μg/mL streptomycin at 37 °C in a humidified incubator with 5% CO_2_. Only passages 0–2 (P0–P2) were used for experiments to maintain the primary phenotype. For identification, cells were fixed and immunostained with a primary antibody against collagen type II (1:200, Abcam) followed by a fluorescent secondary antibody. Images were captured using a fluorescence microscope (Nikon Eclipse Ti).

### 2.3 Cell treatments and transfection

To establish an *in vitro* inflammation model, chondrocytes were treated with 1 μg/mL lipopolysaccharide (LPS, Sigma) for 24 h. For CDK9 inhibition, cells were pretreated with FLA (1, 5, or 10 μM; Selleckchem) for 2 h prior to LPS stimulation. In rescue experiments, the NF-κB activator IL-1β (10 ng/mL; PeproTech) was co-administered with FLA.

To knock down the expression of CDK9 (Gene ID: NM_001007744.1), this study utilized pre-designed siRNA (Product ID: RX053724) provided by Anhui Tongyuan Biotechnology Co., Ltd. for transfection. The siRNA sequences are as follows: si-CDK9-1 (5′to 3′): GGU​CAC​CAA​GUA​CGA​GAA​ATT, UUU​CUC​GUA​CUU​GGU​GAC​CTT; si-CDK9-2 (5′to 3′): GCU​GCU​GAG​CAA​UGU​CUU​ATT, UAA​GAC​AUU​GCU​CAG​CAG​CTT; si-CDK9-3 (5′to 3′): CCU​UCA​GCC​UAG​CUA​AGA​ATT, UUC​UUA​GCU​AGG​CUG​AAG​GTT; si-NC (5′to 3′): UUC​UCC​GAA​CGU​GUC​ACG​UT, ACG​UGA​CAC​GUU​CGG​AGA​ATT.

For gene silencing, chondrocytes were transfected with 50 nM of CDK9-targeting siRNA (si-CDK9-1, −2, −3) or negative control siRNA (si-NC) using Lipofectamine 2000 (Invitrogen). For overexpression, cells were transfected with 2.5 μg of pcDNA3.1-CDK9 plasmid (pc-CDK9) or empty vector (pc-NC). Transfection efficiency was assessed 48 h post-transfection by RT-qPCR and Western blot.

### 2.4 RNA extraction and quantitative real-time PCR (RT-qPCR)

Total RNA was extracted using TRIzol reagent (Invitrogen) and reverse transcribed into cDNA with a PrimeScript RT kit (Takara). RT-qPCR was performed on a Bio-Rad CFX96 system using SYBR Green Master Mix (Yeasen). The primer sequences were: CDK9: F 5′-CAG​GAA​CAA​GAT​CCT​GCA​C-3′, R 5′-CAG​GAA​CAA​GAT​CCT​GCA​C-3′

NF-κB p65: F 5′-GGG​AGA​TGT​GAA​GAT​GCT​G-3′, R 5′-AAG​TGT​AGG​ACA​CTG​TCC​C-3′

MMP-13: F 5′-TTT​CCT​CCT​GGA​CCA​AAC​C-3′, R 5′-AGT​TGT​AGC​CTT​TGG​AGC​T-3′

ADAMTS-5: F 5′-ACA​ACC​AGC​TAG​GTG​ATG​AC-3′, R 5′-AAT​GAT​GCC​CAC​ATA​AAT​CCT​C-3′

β-actin: F 5′-CCT​GGC​ACC​CAG​CAC​AAT-3′, R 5′-GGG​CCG​GAC​TCG​TCA​TAC-3′

Relative mRNA expression was calculated using the 2^−ΔΔCt^ method with β-actin as the internal control.

### 2.5 Western blot analysis

Proteins were extracted using RIPA buffer containing protease and phosphatase inhibitors. Lysates were separated by SDS-PAGE and transferred to PVDF membranes. After blocking with 5% non-fat milk, membranes were incubated overnight at 4 °C with primary antibodies against CDK9, p65, phospho-p65 (Ser536), MMP-13, ADAMTS-5 (all 1:1000, Abcam), IKKα, p-IKKα, IKKβ, p-IKKβ (1:1000, Cell Signaling Technology), and β-actin (1:3000, Abcam). Following incubation with HRP-conjugated secondary antibodies (1:5000, ZSGB-BIO), protein bands were visualized using an ECL kit (Thermo Scientific) and quantified by ImageJ software.

### 2.6 Cell viability and apoptosis assays

Cell viability was assessed using the CCK-8 kit (Beyotime). Chondrocytes were seeded in 96-well plates (1 × 10^4^ cells/well), treated accordingly, and incubated with 10 μL CCK-8 reagent for 4 h. Absorbance was measured at 450 nm using a microplate reader (Thermo MK3). Apoptosis was detected using an Annexin V-APC/7-AAD Apoptosis Detection Kit (TransGen). Cells were harvested, stained according to the manufacturer’s protocol, and analyzed on a NovoCyte flow cytometer (Agilent). Data were processed using NovoExpress software.

### 2.7 Enzyme-linked immunosorbent assay (ELISA)

Levels of TGF-β, TNF-α, and IL-6 in cell culture supernatants or rat synovial fluid were measured using commercial ELISA kits (Ybscience, Shanghai) according to the manufacturer’s instructions. Absorbance was read at 450 nm, and concentrations were calculated from standard curves.

### 2.8 Animal model and drug administration

All animal procedures were approved by the Institutional Animal Care and Use Committee of Nantong University (Approval No. S20230805-099). Sixty male SD rats (8 weeks old) were randomly divided into six groups (n = 10/group): (1) Sham, (2) PTOA (Model), (3) Model + FLA (1 mg/kg), (4) Model + FLA (3.5 mg/kg), (5) Model + FLA (7.5 mg/kg), and (6) Model + FLA (7.5 mg/kg) + IL-1β (10 ng/joint).

PTOA was induced by medial meniscectomy (MMx) under anesthesia. Briefly, the rats were anesthetized by intraperitoneal injection of 3% pentobarbital sodium (30 mg/kg), then a medial parapatellar incision was made, the medial meniscus was exposed and completely excised, and the capsule and skin were sutured. Sham-operated rats underwent the same surgical exposure without meniscus removal. Starting on the day of surgery, rats received intra-articular injections of FLA (prepared in saline with 5% DMSO) or vehicle every other day for a total of 5 injections over 10 days. In group 6, IL-1β was co-injected with FLA. Behavioral tests were conducted 7 days after the last injection. Rats were euthanized with 100% CO_2_ using a gradual-fill method. CO_2_ was introduced into an uncharged chamber at a displacement rate of 30%–70% of the chamber volume per minute, in accordance with current euthanasia guidelines. CO_2_ flow was maintained for ≥1 min after respiratory arrest, and death was confirmed by absence of respiration and heartbeat prior to tissue collection.

### 2.9 Behavioral assessments

The mechanical withdrawal threshold was measured using an electronic von Frey apparatus (IITC Inc.) applied to the plantar surface of the hind paw. Spontaneous locomotor activity was evaluated in an open-field arena (50 × 50 × 40 cm). Each rat was placed in the center and allowed to explore freely for 5 min. Total distance traveled, average speed, and time spent in the central zone were recorded and analyzed using EthoVision XT software (Noldus).

### 2.10 Histopathology and immunohistochemistry

Knee joints were fixed in 4% paraformaldehyde, decalcified in 10% EDTA, paraffin-embedded, and sectioned coronally at 5 μm thickness. Sections were stained with hematoxylin-eosin (HE) or Safranin O-Fast Green for general morphology and proteoglycan content, respectively. Osteoarthritis severity was evaluated using the Mankin score (van der Sluijs et al., 1992) and the OARSI scoring system (Gerwin et al., 2010) by two blinded investigators. For immunohistochemical detection, paraffin sections were baked at 60 °C for 2 h, deparaffinized in xylene, and rehydrated through a graded ethanol series, followed by rinsing with distilled water. Endogenous peroxidase activity was blocked by incubating the sections with 3% H_2_O_2_ for 30 min at room temperature. Antigen retrieval was performed by high-pressure processing in citrate buffer for 2 min using a pressure cooker. After allowing to cool naturally, the sections were washed with PBS and blocked for 30 min. The sections were then incubated overnight at 4 °C with primary antibodies against CDK9, p65, and p-p65, each diluted 1:200 (R&D Systems). Subsequently, HRP-conjugated secondary antibody was applied and incubated for 60 min at 37 °C. After thorough washing with PBS, immunoreactivity was visualized using a DAB chromogen. Sections were counterstained with hematoxylin, dehydrated through a graded ethanol series, cleared in xylene, and mounted with neutral balsam. After drying, the slides were observed under a microscope.

### 2.11 Statistical analysis

Data are presented as mean ± standard deviation. Normality was assessed using the Shapiro-Wilk test. Comparisons between two groups were performed using Student’s t-test. One-way analysis of variance (ANOVA) followed by Tukey’s *post hoc* test was used for multiple group comparisons. A p-value <0.05 was considered statistically significant. All analyses were conducted using GraphPad Prism 9.0.

## 3 Results

### 3.1 CDK9 is upregulated in OA and validated in experimental models

**FIGURE 1:**
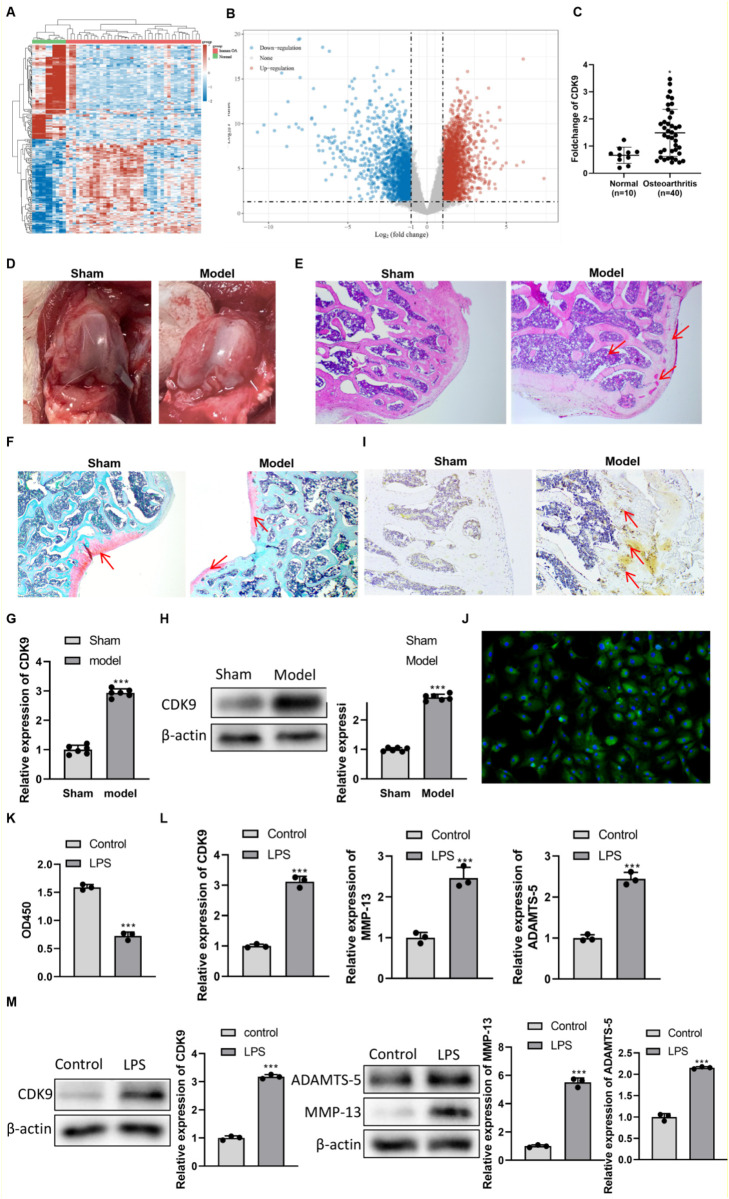
Construction and identification of rat traumatic knee arthritis model *in vivo* and cell model *in vitro*. **(A)** The expression of different genes in each OA and normal knee tissue sample was analyzed by cluster heat map. **(B)** The difference of gene expression in OA and non-OA knee tissues was analyzed by volcano map. **(C)** Expression levels of CDK9 in human OA and normal knee tissues in GSE51588 expression data. **P < 0.01 versus Normal. **(D)** The knee joint condition of rats after 4 weeks of modeling. **(E)** Hematoxylin and eosin (HE) staining of rat knee joint tissue sections. **(F)** Safranine solid green staining of knee tissues (×100). **(G)** CDK9 mRNA expression levels in rat cartilage tissue detected by RT-PCR. ***P < 0.001 versus Sham. **(H)** CDK9 protein levels in rat cartilage tissue detected by western blot. ***P < 0.001 versus Sham. **(I)** Immunohistochemical staining of CDK9 in rat cartilage tissue (×100). **(J)** Identification of primary rat chondrocytes by immunofluorescence staining for collagen type II (green fluorescence). **(K)** Lps-induced cell activity was detected by CCK8 *in vitro*. **(L)** Relative mRNA expression levels expression of CDK9, MMP-13 and ADAMTS-5 was detected by PCR. **(M)** The protein expression levels of CDK9, MMP-13 and ADAMTS-5 was detected by Western Blot *in vitro*. Data are presented as mean ± SD. ***P < 0.001 versus Control.

Bioinformatics analysis of the GSE51588 dataset revealed distinct gene expression profiles between human OA and normal subchondral bone (Figures 1A,B). Notably, CDK9 expression was significantly upregulated in OA tissues (Figure 1C). To experimentally investigate CDK9’s role, we established a rat PTOA model by medial meniscectomy (MMx). Macroscopic examination at 4 weeks post-surgery showed cartilage surface irregularities, sclerosis, and loss of gloss in the model group, contrasting with the smooth, intact cartilage in sham controls (Figure 1D). Histologically, HE staining revealed structural disruption, chondrocyte loss, and fissure formation in the model group (Figure 1E). Safranin O–Fast Green staining demonstrated severe proteoglycan depletion and cartilage matrix degradation in PTOA rats, along with a disrupted cartilage-bone interface (Figure 1F). Consistent with bioinformatics data, both mRNA and protein levels of CDK9 were significantly elevated in the cartilage of model rats compared to sham controls (Figures 1G–I).

Primary rat chondrocytes were successfully isolated and identified by positive collagen type II immunofluorescence (Figure 1J). To mimic inflammatory injury *in vitro*, cells were treated with LPS. LPS stimulation significantly reduced chondrocyte viability (Figure 1K) and markedly increased the expression of CDK9, MMP-13, and ADAMTS-5 at both mRNA and protein levels (Figures 1L,M), confirming the establishment of a cellular OA model.

### 3.2 Modulation of CDK9 expression alters chondrocyte phenotype *in vitro*

**FIGURE 2:**
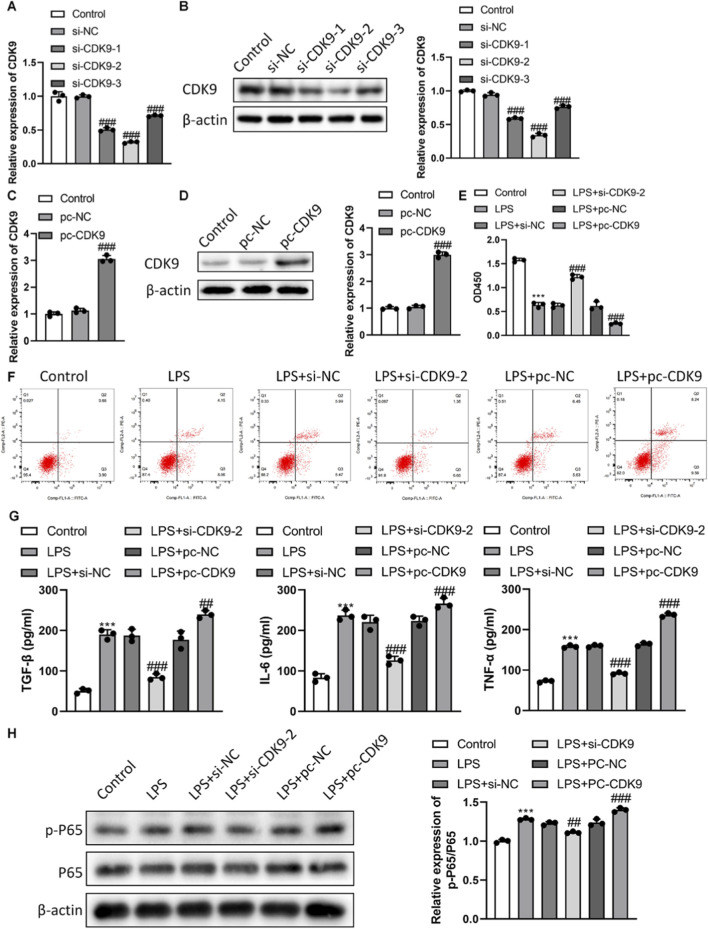
Effect of knockdown/overexpression of CDK9 on chondrocytes *in vitro*. **(A)** CDK9 mRNA knockdown efficiency was evaluated by RT-PCR. **(B)** CDK9 protein knockdown efficiency was evaluated by western blot. **(C)** CDK9 mRNA overexpression efficiency was evaluated by RT-PCR. **(D)** CDK9 protein overexpression efficiency was evaluated by western blot. **(E)** The effect of knockdown/overexpression of CDK9 on cell activity was detected by CCK8. **(F)** The effect of knockdown/overexpression of CDK9 on apoptosis was detected by flow cytometry. **(G)** Levels of TNF-α, IL-6 as well as TGF-β. **(H)** Protein levels of p-p65 and total p65 related to the NF-κB signaling pathway were detected by western blot. Data are presented as mean ± SD (n = 3 per group), ***P < 0.001 versus Control, ###P < 0.001 versus LPS + si-NC/LPS + pc-NC.

We genetically modulated CDK9 levels using siRNA and overexpression plasmids. Transfection with si-CDK9-2 achieved the most efficient knockdown (∼70% reduction at mRNA and protein levels), while pc-CDK9 transfection induced robust overexpression (Figures 2A–D). Functionally, CDK9 knockdown significantly attenuated the LPS-induced reduction in cell viability, whereas CDK9 overexpression exacerbated it (Figure 2E). Similarly, CDK9 silencing markedly decreased LPS-triggered apoptosis, while overexpression further increased apoptotic rates (Figure 2F). ELISA analysis showed that CDK9 knockdown suppressed the LPS-induced secretion of pro-inflammatory cytokines (TGF-β, TNF-α, IL-6), whereas overexpression enhanced their release (Figure 2G). At the signaling level, CDK9 knockdown inhibited LPS-induced phosphorylation of p65 (a key NF-κB subunit), while overexpression enhanced it (Figure 2H), suggesting CDK9 regulates chondrocyte inflammation and survival via the NF-κB pathway.

### 3.3 Pharmacological inhibition of CDK9 protects chondrocytes *in vitro*

**FIGURE 3:**
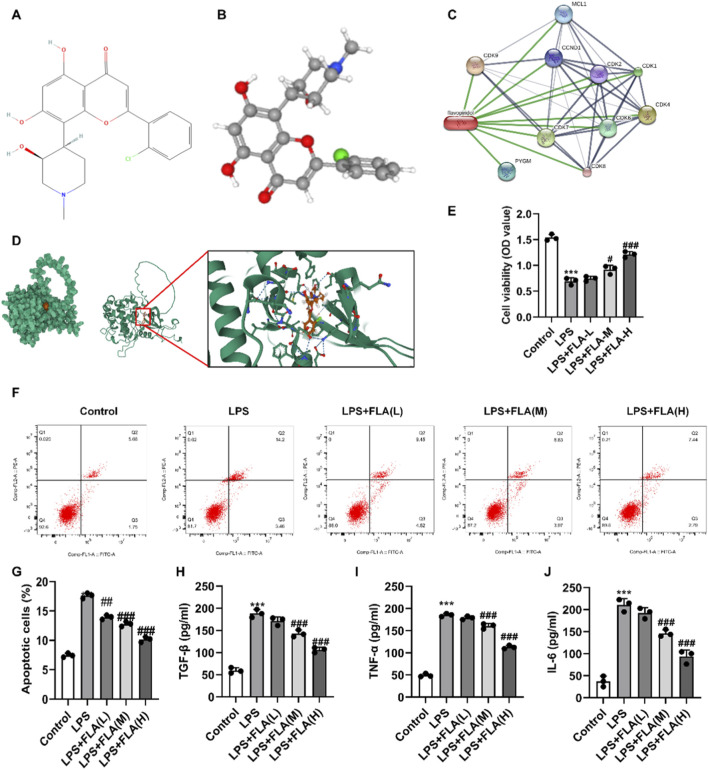
Effect of inhibition CDK9 on the activity and apoptosis of LPS-treated chondrocytes. **(A)** Structural formula of Flavopiridol. **(B)** 3D structure of Flavopiridol. **(C)** FLA-protein interaction network. **(D)** The binding mode of FLA and CDK9. **(E)** The effect of flavopyriol on the activity of LPS-treated chondrocytes was detected by CCK8. **(F,G)** Apoptosis of chondrocytes measured by flow cytometry using Annexin V/PI staining. **(H–J)** The levels of inflammatory factors TGF-β, TNF-α and IL-6 in the cell culture supernatants were detected by Elisa. Data are presented as mean ± SD (n = 3 per group). ***P < 0.001 versus Control, ##P < 0.01, ###P < 0.001 versus LPS.

Subsequently, we employed the pharmacological CDK9 inhibitor flavopiridol (FLA) (Figures 3A,B). The three-dimensional structure of FLA was obtained from the PubChem database. Network analysis (STITCH) and molecular docking were performed to predict the potential interaction between FLA and CDK9. Molecular docking was carried out to verify the binding capacity at the molecular structural level, and the binding affinity between FLA and CDK9 was determined to be −11.34 kcal/mol, supporting a strong and stable interaction (Figures 3C,D). FLA treatment dose-dependently restored cell viability in LPS-stimulated chondrocytes (Figure 3E). Correspondingly, FLA significantly and dose-dependently reduced LPS-induced apoptosis (Figures 3F,G). Furthermore, FLA potently inhibited the LPS-stimulated secretion of TGF-β, TNF-α, and IL-6 (Figures 3H–J). These results confirm that pharmacological CDK9 inhibition replicates the protective effects of genetic knockdown.

### 3.4 CDK9 inhibition alleviates disease severity in a rat PTOA model

**FIGURE 4:**
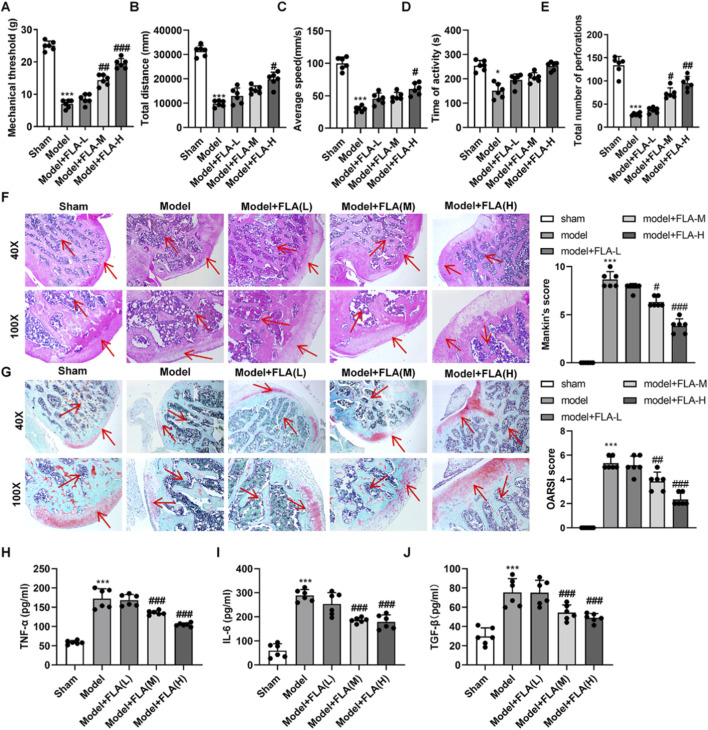
Effect of inhibition or overexpression of CDK9 in vivo on OA. **(A)** Mechanical pain threshold of rats was measured by Von Frey filament test. **(B–E)** Locomotor activity of rats was assessed by open field test, including total distance traveled and movement speed **(F)** Histological examination of knee joint tissues by hematoxylin and eosin staining. **(G)** Safranin O–fast green staining of knee joint tissues to evaluate cartilage degradation. **(H–J)** Levels of TNF-α, IL-6 as well as TGF-β. Data are presented as mean ± SD (n = 6 per group), *P < 0.05, ***P < 0.001 versus Sham, #P < 0.05, ##P < 0.01, ###P < 0.001, versus Model.

PTOA rats were treated with intra-articular FLA at three doses (1, 3.5, 7.5 mg/kg). FLA treatment, particularly at the high dose (7.5 mg/kg), significantly restored the mechanical withdrawal threshold that was reduced in the model group (Figure 4A). In the open field test, high-dose FLA improved locomotor activity, increasing total distance, average speed, and exploratory behavior (Figures 4B–E). Histopathological analysis revealed that FLA treatment ameliorated cartilage structure, reduced chondrocyte death, and attenuated proteoglycan loss in a dose-dependent manner (Figures 4F,G). Consistently, FLA (medium and high doses) significantly lowered the elevated levels of TNF-α, IL-6, and TGF-β in the synovial fluid of PTOA rats (Figures 4H–J).

### 3.5 The protective effect of CDK9 inhibition is mediated through the NF-κB pathway

**FIGURE 5:**
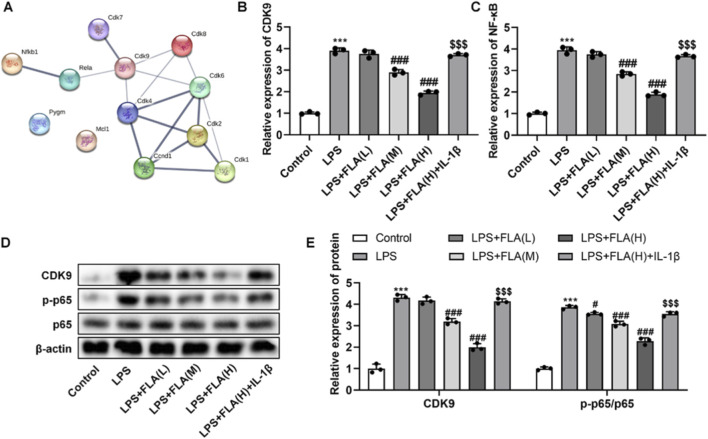
The association of NF-κB pathway with inhibition of CDK9 treatment of chondrocytes was found *in vitro*. **(A)** Interaction network diagram of CDK9-associated protein and NF-κB protein. **(B)** Relative mRNA expression level of CDK9 in LPS-treated chondrocytes detected by PCR. **(C)** Relative mRNA expression level of NF-κB (p65) in LPS-treated chondrocytes detected by RT-qPCR **(D,E)** Protein expression levels of CDK9, total p65 and phosphorylated p65 in chondrocytes were determined by Western blot analysis. Data are presented as mean ± SD (n = 3 per group), ***P < 0.001 versus Control, #P < 0.05, ###P < 0.001 versus LPS, $$$P < 0.001 versus LPS + FLA(H).

**FIGURE 6:**
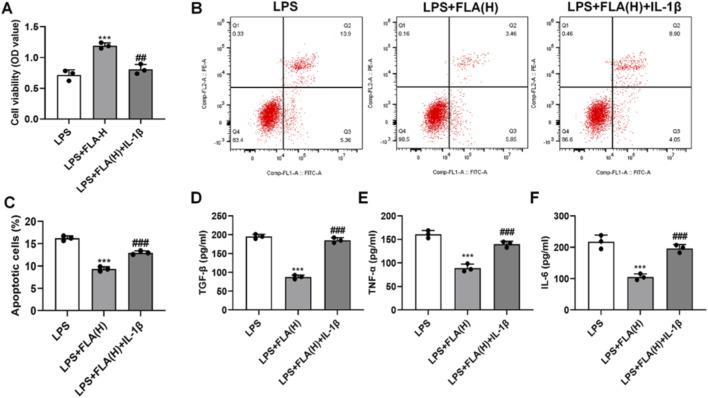
Effect of CDK9/NF-κB signaling pathway on the activity and apoptosis of chondrocytes. **(A)** Cell viability of chondrocytes was assessed using the CCK-8 assay. **(B,C)** Apoptosis of chondrocytes was evaluated by flow cytometry analysis. **(D–F)** The protein levels of inflammatory cytokines TNF-α, IL-6, and TGF-β in the cell culture supernatant were measured by ELISA. Data are presented as mean ± SD (n = 3 per group), ***P < 0.001 versus LPS, ##P < 0.01 versus LPS + FLA(H).

Protein interaction network analysis (STRING) indicated an association between CDK9 and components of the NF-κB pathway (Figure 5A). To test functional involvement, we co-treated cells with FLA and the NF-κB activator IL-1β. IL-1β treatment effectively reversed the FLA-mediated downregulation of CDK9 and NF-κB (p65) mRNA (Figures 5B,C) and the inhibition of p65 phosphorylation (Figures 5D,E). Moreover, IL-1β abolished the protective effects of FLA on cell viability (Figure 6A) and apoptosis (Figures 6B,C), and re-induced the secretion of inflammatory cytokines (Figures 6D–F).

**FIGURE 7:**
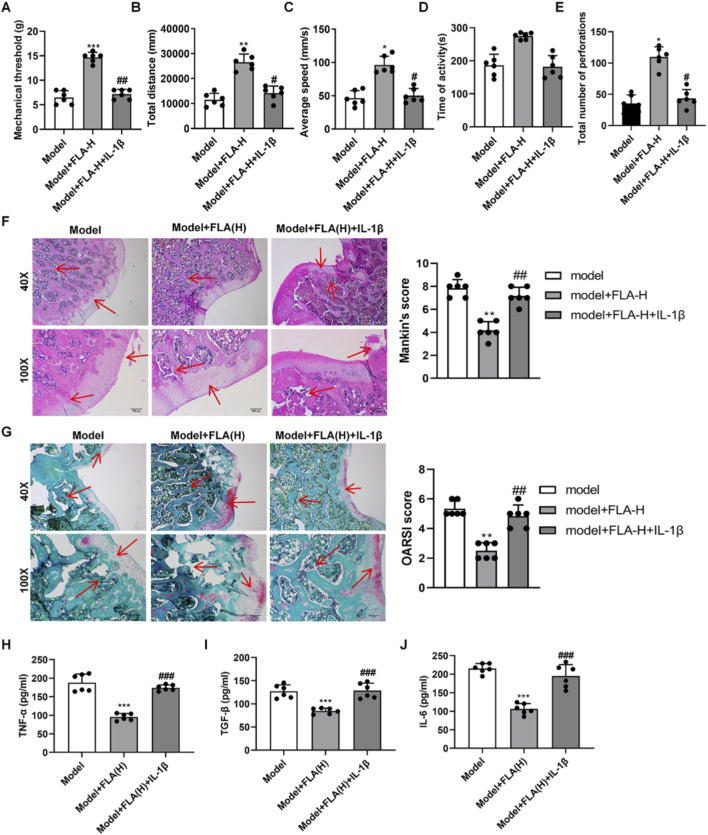
*In vivo* validated inhibition of CDK9 in the treatment of PTOA via the NF-κB pathway. **(A)** Mechanical withdrawal threshold of rats was assessed using a von Frey apparatus. **(B–E)** Activity of rat in open field. **(F)** Histopathological changes in the knee joint cartilage were evaluated by hematoxylin and eosin staining. **(G)** Safranine solid green staining of knee tissues. **(H–J)** Levels of TNF-α, IL-6 as well as TGF-β. Data are presented as mean ± SD (n = 6 per group), *P < 0.05, **P < 0.01 ***P < 0.001 versus Model, #P < 0.05, ##P < 0.01 versus Model + FLA(H).

**FIGURE 8:**
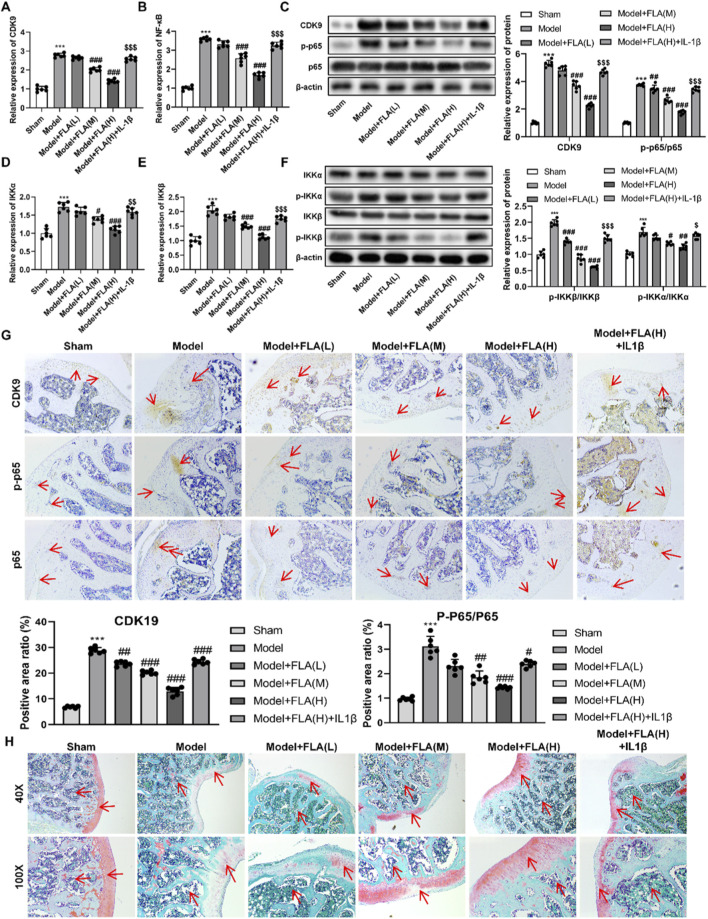
Inhibition of CDK9 *in vivo* affects traumatic knee arthritis by affecting NF-κB. **(A)** mRNA expression level of CDK9 in knee joint tissues was detected by RT-qPCR. **(B)** mRNA expression level of NF-κB was detected by RT-qPCR. **(C)** Protein expression levels of CDK9, p-p65, and p65 were analyzed by Western blotting. **(D)** mRNA expression of IKKα was determined by RT-qPCR. **(E)** mRNA expression of IKKβ was determined by RT-qPCR. **(F)** Protein expression levels of p-IKKα, IKKα, p-IKKβ and IKKβ were measured by Western blotting. **(G)** Immunohistochemistry of CDK9, p-p65 as well as p65 (×40). **(H)** Safranine solid green staining of cartilage sections. Data are presented as mean ± SD (n = 6 per group), ***P < 0.001 versus Sham, #P < 0.05, ##P < 0.01, ###P < 0.001 versus Model, $P < 0.05, $$P < 0.01, $$$P < 0.001 versus Model + FLA(H).

*In vivo*, IL-1β co-administration similarly reversed the therapeutic benefits of high-dose FLA. It blunted the improvement in pain threshold and locomotor activity (Figures 7A–E), worsened cartilage histopathology (Figures 7F,G), and elevated synovial cytokine levels (Figures 7H–J). Mechanistically, FLA inhibited the mRNA expression and phosphorylation of key NF-κB pathway kinases IKKα and IKKβ in cartilage tissue, effects that were reversed by IL-1β (Figures 8A–F). Immunohistochemistry confirmed that FLA reduced the abundance of CDK9 and phospho-p65 in cartilage, which was increased upon IL-1β treatment (Figure 8G). Safranin O staining corroborated that IL-1β counteracted FLA’s protection against matrix degradation (Figure 8H).

## 4 Discussion

PTOA is a debilitating sequela of joint injury with limited disease-modifying treatments. This study identifies CDK9 as a novel promoter of PTOA pathogenesis and demonstrates that its inhibition, genetically or pharmacologically, alleviates disease progression primarily by suppressing the NF-κB signaling pathway.

We first confirmed significant upregulation of CDK9 in human OA tissue and in our rat PTOA model, aligning with its emerging role in inflammatory diseases (Fukui et al., 2021). Using multiple approaches—siRNA, overexpression, and the inhibitor FLA—we established a causal relationship between CDK9 activity and chondrocyte dysfunction. CDK9 inhibition enhanced cell viability, reduced apoptosis, and dampened inflammatory cytokine production *in vitro*, and ameliorated pain, functional impairment, and cartilage degradation *in vivo*. These findings extend the known functions of CDK9 beyond transcriptional regulation and cancer (Huang Z. et al., 2022; Mandal et al., 2021) to include a central role in cartilage homeostasis and OA pathogenesis.

A key mechanistic insight from our study is the identification of the NF-κB pathway as a critical downstream effector of CDK9 in chondrocytes. NF-κB is a master regulator of inflammation and cell survival in OA (Choi et al., 2019; Williams and Gilmore, 2020). We found that CDK9 inhibition suppressed p65 phosphorylation and the expression of NF-κB-dependent catabolic (MMP-13, ADAMTS-5) and inflammatory (TNF-α, IL-6, TGF-β) genes. Most importantly, the protective effects of FLA were completely abolished by the NF-κB activator IL-1β in both models, providing strong genetic and pharmacological evidence for a CDK9–NF-κB axis in PTOA.

Our work advances the field by delineating a more upstream regulatory mechanism. While previous studies noted NF-κB activation in OA (Mulero et al., 2019), we show that CDK9 inhibition suppresses the phosphorylation of IKKα and IKKβ, the key kinases responsible for NF-κB activation. This suggests CDK9 may regulate the NF-κB pathway at the level of the IKK complex, offering a novel mechanistic link not previously reported in PTOA. This distinction adds significant depth to earlier studies that focused on CDK9 inhibition for PTOA without fully elucidating its signaling cascade (Fukui et al., 2021; Sangsuwan et al., 2022; Hu et al., 2016).

The therapeutic potential of CDK9 inhibition is underscored by the efficacy of FLA, a first-in-class CDK inhibitor (Pawde et al., 2024; Senderowicz, 1999). Our dose-dependent findings and rescue experiments suggest that targeting CDK9 could be a viable strategy to modulate the inflammatory microenvironment in injured joints. However, the reversal by IL-1β highlights the complexity and resilience of the inflammatory network in PTOA, implying that combination therapies targeting multiple pathways might be necessary for sustained efficacy.

Notably, CDK9 plays distinct roles in different arthritides due to pathological differences. In PTOA, it mediates acute trauma-induced inflammatory gene transcriptional bursts, with targeted inhibition focusing on early preemptive intervention. In RA, it regulates chronic autoimmune inflammation and synovial fibroblast/osteoclast proliferation, offering immunomodulatory and anti-proliferative potential. In primary OA, it modulates SASP and low-grade inflammation. Currently, CDK9-targeted drugs are in RA clinical trials, but PTOA/OA research remains preclinical—an unmet gap our study addresses.

Compared with existing therapies, CDK9 inhibitors offer broad-spectrum anti-inflammatory effects by targeting upstream inflammatory hubs, blocking multiple pathogenic factors. However, clinical translation faces challenges: short half-lives of small-molecule inhibitors (needing sustained-release delivery), potential cardiotoxicity immunosuppression with long-term use, and the need for PTOA’s therapeutic “golden window” and RA patient stratification. Most CDK9 inhibitors are developed for oncology, with limited arthritis research—emphasizing further exploration.

Our study has limitations. First, while FLA is a potent CDK9 inhibitor, its off-target effects on other CDKs cannot be entirely ruled out, though the concordance with siRNA data strengthens specificity. Second, the use of a young, male rat model may not fully replicate the complexity of human PTOA, which often involves aging, metabolic factors, and hormonal influences (Blaker et al., 2017). Future studies should validate these findings in aged or female animals and in human OA chondrocytes. Third, the specific molecular step by which CDK9 regulates IKK phosphorylation warrants further investigation—whether through direct phosphorylation, interaction with regulatory subunits, or modulation of upstream kinases. Finally, long-term safety and efficacy studies of CDK9 inhibition in articular joints are needed before clinical translation.

In summary, our integrated approach demonstrates that CDK9 drives inflammation and chondrocyte apoptosis in PTOA. Inhibition of CDK9, via suppression of the IKK–NF-κB pathway, confers significant protection in preclinical models. These findings establish CDK9 as a promising and novel therapeutic target for the development of disease-modifying therapies against post-traumatic osteoarthritis.

## 5 Conclusion

In conclusion, our study comprehensively demonstrates that downregulating CDK9 expression alleviates pain and activity impairment in PTOA rats by inhibiting the NF-κB signaling pathway. In turn, reducing the inflammatory response. To validate these vitro experiments, corroborated these findings. Specifically, inhibiting CDK9 expression and suppressing the NF-κB pathway restores knee joint chondrocyte viability, leading to a reduction in both apoptosis and inflammation. These results suggest that CDK9 holds promise as a potential therapeutic target for the clinical treatment of PTOA.

## Data Availability

Publicly available datasets were analyzed in this study. This data can be found here: https://www.ncbi.nlm.nih.gov/geo/query/acc.cgi?acc&equals;GSE51588.
